# RNA secondary structure and nucleotide composition of the conserved hallmark sequence of *Leishmania* SIDER2 retroposons are essential for endonucleolytic cleavage and mRNA degradation

**DOI:** 10.1371/journal.pone.0180678

**Published:** 2017-07-13

**Authors:** Hiva Azizi, Tatiany P. Romão, Karen Santos Charret, Prasad K. Padmanabhan, Osvaldo P. de Melo Neto, Michaela Müller-McNicoll, Barbara Papadopoulou

**Affiliations:** 1 Research Center in Infectious Diseases, CHU de Quebec Research Center-University Laval, Quebec, QC. Canada; 2 Department of Microbiology-Infectious Disease and Immunology, Faculty of Medicine, University Laval, Quebec, QC. Canada; 3 Departamento de Microbiologia, Centro de Pesquisas Aggeu Magalhães-FIOCRUZ, Recife, PE, Brazil; 4 Fundação Oswaldo Cruz-FIOCRUZ, Rio de Janeiro, Brazil; 5 RNA Regulation Group, Cluster of Excellence ‘Macromolecular Complexes’, Goethe-University Frankfurt, Institute of Cell Biology and Neuroscience, Frankfurt /Main, Germany; University of Cambridge, UNITED KINGDOM

## Abstract

We have reported previously that **S**hort **I**nterspersed **D**egenerate **R**etroposons of the SIDER2 subfamily, largely located within 3'UTRs of *Leishmania* transcripts, promote rapid turnover of mRNAs through endonucleolytic cleavage within the highly conserved second tandem 79-nt hallmark sequence (79-nt SII). Here, we used site-directed mutagenesis and *in silico* RNA structural studies to delineate the *cis*-acting requirements within 79-nt SII for cleavage and mRNA degradation. The putative cleavage site(s) and other nucleotides predicted to alter the RNA secondary structure of 79-nt SII were either deleted or mutated and their effect on mRNA turnover was monitored using a gene reporter system. We found that short deletions of 8-nt spanning the two predicted cleavage sites block degradation of SIDER2-containing transcripts, leading to mRNA accumulation. Furthermore, single or double substitutions of the dinucleotides targeted for cleavage as well as mutations altering the predicted RNA secondary structure encompassing both cleavage sites also prevent mRNA degradation, confirming that these dinucleotides are the *bona fide* cleavage sites. In line with these results, we show that stage-regulated SIDER2 inactivation correlates with the absence of endonucleolytic cleavage. Overall, these data demonstrate that both cleavage sites within the conserved 79-nt SII as well as RNA folding in this region are essential for SIDER2-mediated mRNA decay, and further support that SIDER2-harboring transcripts are targeted for degradation by endonucleolytic cleavage.

## Introduction

*Leishmania* species cause a broad spectrum of vector-borne parasitic diseases, ranging from self-healing cutaneous to fatal visceral forms that are collectively termed as leishmaniasis. The disease has expanded to over 98 countries with more than 350 million people at risk and 2 million individuals infected annually [1, 2]. *Leishmania* is an early-branching unicellular eukaryote [3] that belongs to the *Trypanosomatidae* family. These organisms lack defined RNA pol II promoters and the typical general transcription factors and have most likely lost the ability to regulate gene expression at the level of transcription initiation. Instead, RNA polymerase II transcribes long polycistronic units that are further processed into individual mature mRNAs by two coupled RNA-processing reactions, namely, *trans*-splicing which adds the 39-nt spliced leader RNA to the 5' terminus of all protein-encoding RNAs and 3'-polyadenylation [4, 5]. Thus, regulation of gene expression in these organisms occurs exclusively at the post-transcriptional level [6, 7].

*Cis*-acting motifs within 3'UTRs of *Leishmania* transcripts have been shown to play major roles in regulating mRNA stability and translation [8–15]. We have previously identified and characterized a large class of *cis*-acting elements in *Leishmania*, SIDERs for **S**hort **I**nterspersed **DE**generate **R**etroposons [11]. SIDERs are truncated versions (~0.55 kb) of formerly active retroposons of the *ingi*/L1Tc-related family that are predominantly located (>75%) in 3'UTRs with >20% of the *Leishmania* transcripts bearing SIDER elements [11, 16, 17]. SIDERs constitute the largest family of transposable elements described so far in trypanosomatid genomes and are sub-divided into two phylogenetically distinct subfamilies, SIDER1 and SIDER2. Members of the SIDER1/2 subfamilies were shown to regulate mostly mRNA turnover or translation in a stage- and species-specific manner [8, 9, 11, 13, 14, 18, 19] and possibly form RNA regulons [14, 20]. SIDERs also contribute to genome plasticity through homologous recombination events in response to diverse environmental stimuli such as drug pressure [21].

We have shown previously that retroposons of the SIDER2 subfamily promote rapid mRNA turnover through endonucleolytic cleavage without prior deadenylation [14] unlike most eukaryotic transcripts whose decay begins with poly(A) shortening [22]. Cleavage of SIDER2-containing transcripts was mapped within the second tandem 79-nt hallmark sequence (79-nt Signature II; SII) [14], which represents the most conserved sequence among SIDER2 retroposons [11, 17]. Deletion of either the whole SIDER2 or the 79-nt SII blocked endonucleolytic cleavage and resulted in transcript accumulation [14]. The widespread distribution of SIDER2 retroposons in 3'UTRs of *Leishmania* mRNAs [17] and their proven role in regulating mRNA stability qualifies these *cis*-acting elements as major regulators of post-transcriptional control in these parasites.

In this study, we investigated the sequence and structural requirements within the second 79-nt hallmark sequence of SIDER2 retroposons for efficient mRNA cleavage and degradation using mutational analysis and *in silico* RNA structural studies. We introduced small deletions or single and double nucleotide substitutions within 79-nt SII to modify the putative cleavage site(s) and/or to alter the predicted secondary structure and examined their effect on mRNA turnover. We show that mutations altering the predicted cleavage sites as well as RNA structure encompassing this region block mRNA degradation, indicating that rapid mRNA turnover by SIDER2 elements is strictly dependent on initial cleavage and that it requires a specific RNA secondary structure. Furthermore, we show that stage-specific accumulation of SIDER2-containing transcripts correlates with the absence of endonucleolytic cleavage products. Together these data clearly support a model of endonucleolytic cleavage being central to the mechanism of SIDER2-mediated decay in *Leishmania*.

## Materials and methods

### *Leishmania* culture

*Leishmania major* LV39 and *L*. *infantum* MHOM/MA/67/ITMAP-263 strains used in these studies have been described previously [23]. Axenic promastigotes were cultured in SDM-79 medium, supplemented with 10% heat-inactivated FCS (Wisent), 5 μg ml^−1^ hemin at pH 7.0 and 25°C. To obtain *L*. *infantum* axenic amastigotes, stationary-phase promastigotes were inoculated into MAA-20 medium supplemented with 20% FCS and grown at pH 5.6 and 37°C in a 5% CO_2_ [23].

### DNA constructs and transfections

Plasmids generated in this study used the parental vector pSP72-YNEOαLUC described elsewhere [8]. To make the pSP72-YNEOαLUC-3810ΔC11 or pSP72-YNEOαLUC-3810ΔCl2 plasmids in which the previously mapped cleavage site 1 or 2 within the *L*. *major* LmjF.36.3810 SIDER2 [14] were deleted, respectively, the LmjF.36.3810 3ʹUTR was amplified by Taq DNA polymerase (Qiagen) in two separate halves using primers containing BamHI and HindIII, so that 8 nucleotides including the cleavage site 1 or 2 were omitted during the overlapping PCR reaction. To engineer plasmids carrying point mutations (M1 to M6 and M6-1 and M6-2) in the 3810-SIDER2 signature II (SII) sequence, we amplified the 3810–3ʹUTR using the same set of 5ʹ and 3ʹ end primers (see S1 Table) in two parties along with primers specific to each mutation. All mutants were generated by amplifying upstream and downstream fragments containing the mutated nucleotide(s) that were fused via overlapping PCR and cloned downstream of the luciferase gene (*LUC*) in pSP72-YNEOα-LUC. The mutations were placed in the internal primers, resulting in the replacement of the wild type nucleotides during the overlapping PCR. Details about the primers and restriction enzymes used to generate these constructs are found in S1 Table. The plasmids expressing LUC-3810 3ʹUTR or LUC-3810ΔSI+II have been described before [14]. To integrate the neomycin phosphotransferase gene (*NEO*) into the *L*. *infantum* LinJ.36.4000 locus through homologous recombination, 500 bp of the 5ʹ and 3ʹ sequences flanking the LinJ.36.4000 ORF were fused to the *NEO* ORF. The three fragments, 5ʹUTR-4000, NEO and 3ʹUTR-4000 were tandemly arranged and amplified by the Phusion High Fidelity Taq DNA polymerase (Thermo Scientific). The corresponding band (~1.8 kb) was excised from an agarose gel and purified using the gel extraction kit (Sigma). All constructs have been verified by sequencing. Approximately 10 μg of purified plasmid DNAs (Sigma) or 2 μg of the linear fragments were transfected into *Leishmania* by electroporation as described previously [24]. Stable transfectants were selected and maintained in culture with 0.04 mg/ml G-418 (Sigma) for episomal transfections or 0.01 mg/ml for the integrated cassette.

### DNA, RNA and protein manipulation

Genomic DNA and total RNA of *Leishmania* promastigotes or axenic amastigotes were extracted using the DNAzol and TRIzol (Life Technologies) reagents, respectively following the manufacturer’s instructions. Southern and Northern blot hybridizations were performed following standard procedures. DNA probes were radioactively labeled with dCTP [alpha-32P] using random oligonucleotides and Klenow fragment DNA polymerase I (New England Biolabs). Copy number values for the LUC-3810 3ʹUTR wild type and mutated plasmids as well as for the LUC-3810ΔSI+II vector were estimated following Southern blot hybridization of total DNA extracted from all the different transfectants and digested with NdeI using a probe complementary to the first 1000 nucleotides of the LmJF.36.3810 3ʹUTR, recognizing both the plasmid and the genomic copies. The ratio between the plasmid and genomic copies based on hybridization intensity signals determines the copy number of the different plasmids in all transfectants (see S1 Fig). NEO-expressing *L*. *infantum* promastigotes and third passage axenic amastigotes were used to extract total proteins for Western blotting. Briefly, parasites were first washed twice with phosphate-buffered saline (PBS), quickly centrifuged and lysed in 2 × Laemmli buffer. To evaluate NEO expression, the blots were first reacted with a rabbit anti-NEO antibody at 1:2000 (Millipore) and then with an anti-rabbit-HRP antibody (GE Healthcare). The mouse anti-alpha-tubulin antibody (Sigma) followed by an anti-mouse-HRP antibody (Cell Signalling) was used to normalize for protein loading variations. Blots were visualized by chemoluminescence with Pierce ECL2 Western blotting kit (Thermo Scientific). Protein levels were quantified by densitometric analyses using the ImageQuant 5.2 software.

### Primer extension

Primer extension (PE) was used to detect extended cleavage fragments in recombinant *L*. *major* LUC-3810 3ʹUTR and LUC-3810 SIDER2 as well as in *L*. *infantum* expressing LUC-40003ʹUTR in both life stages essentially as described previously [14]. Primer P1 (20-nt; 5’-GCACAGGCCTGCTCACTGTC-3’) described before [14], complementary to a region at ~150-nt downstream of the first predicted cleavage site in 3810-SIDER2 was used in this study. Briefly, P1 was labeled at the 5'-end with [γ-^32^P]ATP using polynucleotide kinase (PNK; New England Biolabs). Total RNA was isolated by TRIzol and quantified by NanoDrop ND-1000. Approximately 10 pmol of labeled primer and 50 μg of total RNA were used for PE reactions using the SuperScript III RT kit (Invitrogen) according to the manufacturer’s recommendations. A ØX174 DNA/HinfI dephosphorylated DNA marker (Promega) was labeled with [γ-^32^P] ATP and PNK (New England Biolabs) and used as a size marker. Primer extension fragments and markers were separated on 8% denaturating acrylamide gels (Sequagel, National Diagnostics) and visualized by autoradiography.

### RNA structure predictions

To predict the RNA secondary structure of the wild type LmjF.36.3810 SIDER2 79-nt signature II sequence and the different mutants, we used RNAfold [25], one of the core programs of the Vienna RNA package that can predict the minimum free energy (MFE) secondary structure of single-stranded RNA sequences using the dynamic programming algorithm originally proposed by Zuker and Stiegler [26]. In addition to MFE folding, equilibrium base-pairing probabilities were calculated via the John McCaskill's partition function (PF) algorithm [27].

## Results

### Deletion of the predicted cleavage sites within the second 79-nt hallmark sequence of SIDER2 retroposons prevents degradation of SIDER2-containing transcripts

**Fig 1:**
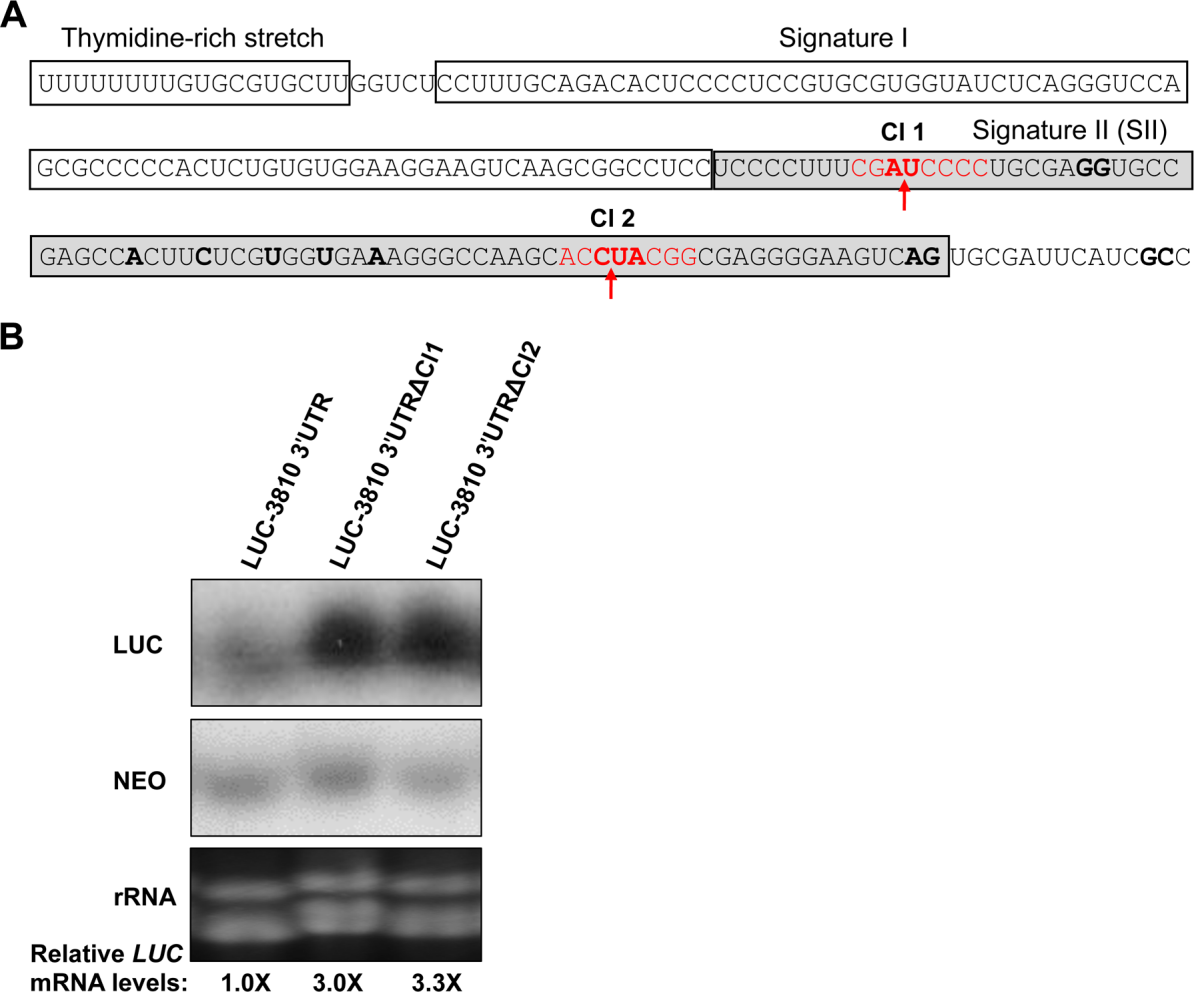
Deletion of the predicted cleavage sites within the second 79-nt hallmark sequence of SIDER2 retroposons prevents SIDER2-mediated mRNA decay. (A) SIDER2 retroposons are generally composed by three conserved motifs; a 18-nt thymidine-rich stretch at the 5'-end corresponding to the former recognition site for an endonuclease encoded by autonomous retroposon elements followed by two tandemly 79-nt sequences (signatures I and II) representing the hallmark of trypanosomatid retroposons. Here the nucleotide sequence of the conserved motifs of the *L*. *major* LmjF.36.3810 SIDER2 element present in the 3'UTR is shown. Deletions of 8-nt flanking the two putative cleavage sites (Cl1 and Cl2) (in red) mapped previously within the second signature sequence of the SIDER2-containing LmjF.36.3810 3'UTR [14]. (B) Northern blot hybridization of total RNA isolated from *L*. *major* LUC-3810 3'UTR and the deletion mutants LUC-3810 3'UTRΔCl1 and LUC-3810-3'UTRΔCl2 with a LUC-specific radiolabeled probe to assess the effect of cleavage 1 and 2 sites and surrounding nucleotide deletions on *LUC* mRNA decay. The blot was also hybridized with a neomycin phosphotransferase (NEO) probe to normalize for plasmid copy number and RNA loading. An ethidium bromide staining visualizing rRNA is also shown as an additional loading control. Normalized LUC-3810 3'UTRΔCl1 and LUC-3810-3'UTRΔCl2 mRNA levels relative to the full length LUC-3810 3'UTR (control) are shown below the blot. Hybridization signals were quantified by the ImageQuant 5.2 software and *LUC* mRNA values were normalized to the *NEO* mRNA. The Northern blots shown here are representative of two independent experiments yielding comparable results.

Generally, SIDER2 retroposons share three conserved motifs. These include an ~18-nt thymidine-rich stretch at the 5'-end corresponding to the former recognition site for an endonuclease encoded by the autonomous DIRE elements followed by two tandemly arranged boxes of 79-nt each (signatures I and II), representing the hallmark of trypanosomatid retroposons (Fig 1A) [11]. We have demonstrated previously that SIDER2-mediated mRNA decay is initiated by a site-specific endonucleolytic cleavage within the second tandem 79-nt hallmark sequence (79-nt SII) that is conserved among all SIDER2 retroposons [14]. Preferential cleavage sites were mapped between pyrimidine (AU) or pyrimidine-pyrimidine (CU) dinucleotides. The first cleavage site (Cl1) was mapped at the 5'-extremity of 79-nt SII between an AU dinucleotide flanked by a 5'-UCCCC-3' duplicated motif and Cl2 between a CU (or a UA) dinucleotide at ~50 nucleotides downstream (3'-end of 79-nt SII) (Fig 1A and [14]). To assess the importance of these predicted cleavage sites in conferring mRNA degradation, we first generated two deletion constructs lacking 8 nucleotides surrounding either the putative Cl1 (AU) or Cl2 (CU) (Fig 1A) from the 3'UTR of the *L*. *major* LmjF.36.3810 (3810) SIDER2-bearing transcript. The truncated 3810 3'UTR was fused downstream of the luciferase (*LUC*) reporter gene as part of the pSPYNEO-alphaIR expression vector and transfected into the *L*. *major* LV39 strain. Stable transfectants were analyzed by Northern blot hybridization to assess the effect of either deletion on *LUC* mRNA turnover. Deletion of either cleavage site blocked degradation and resulted in 3.0- and 3.3-fold accumulation for LUC-3810ΔCl1 and LUC-3810ΔCl2 transcripts, respectively (Fig 1B). These values are similar to those obtained when deleting the entire 79-nt SII from the 3810 3'UTR [14]. Taken together, these data confirm that the previously mapped cleavage sites are essential for RNA destabilization and further support the model of endonucleolytic cleavage for rapid turnover of SIDER2-containing transcripts.

### Mutations within the dinucleotide cleavage sites and or alterations in the predicted RNA secondary structure of the second 79-nt hallmark sequence of SIDER2 retroposons block mRNA decay

**Fig 2:**
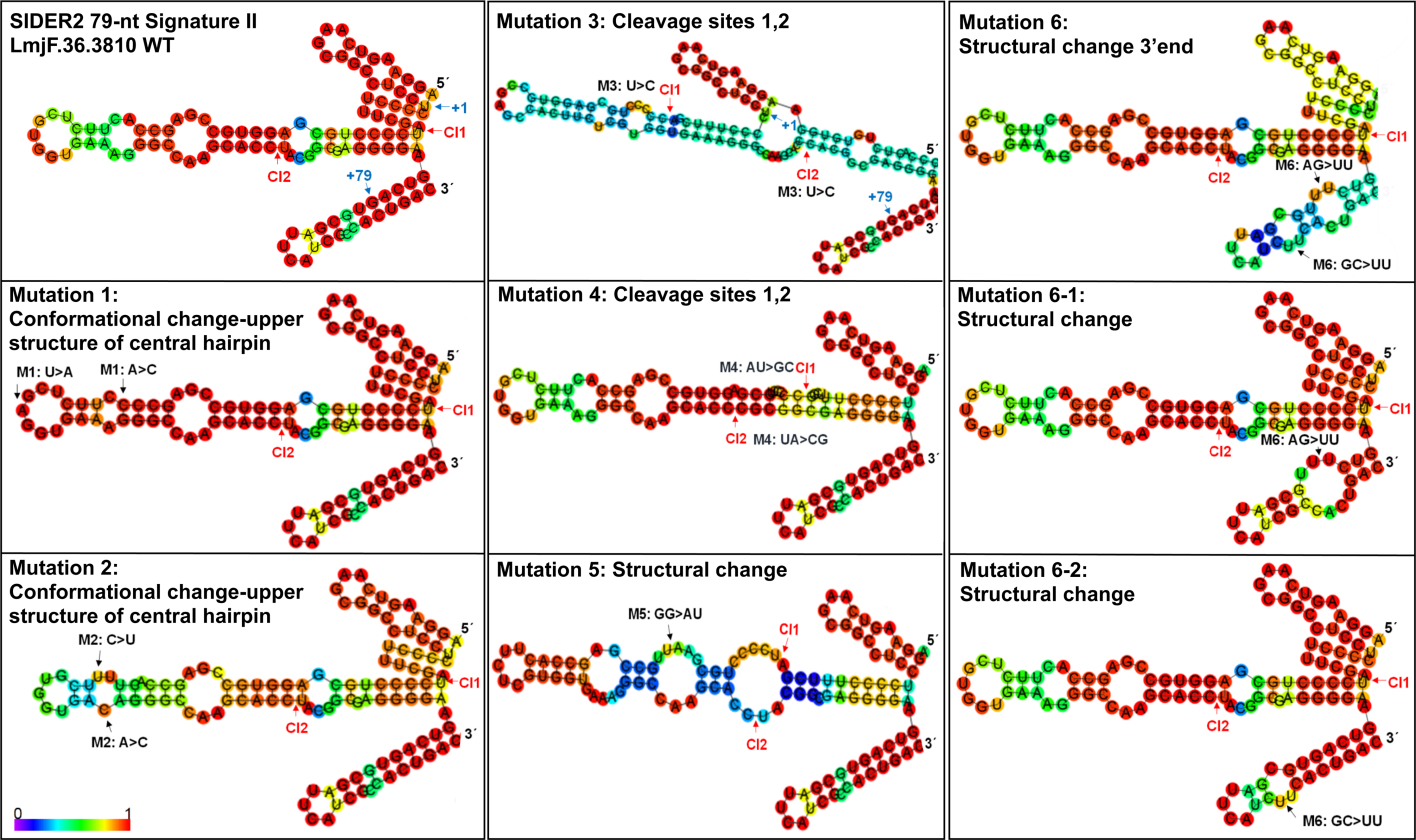
RNA secondary structure predictions of the wild type and mutated versions of the second tandem 79-nt signature sequence of *L*. *major* LmjF.36.3810 SIDER2. The RNAfold web server according to Minimum Free Energy (MFE) was used for RNA secondary structure predictions. Colors in the structures indicate base-pairing probabilities (scale 0–1; 0: magenda, 1: red). Mutant M1 carries two substitutions; an A/C mutation at position +33 and a U/A at +41 nucleotides from the 5'end of 79-nt SII. M2 contains a C/U mutation at +39 and an A/C at +47. M3 encompasses two mutations, a U/C at position +12 and a U/C at +61 within the dinucleotide cleavage sites that have been mapped previously in LmjF.36.3810 SIDER2 [14]. M4 harbors two dinucleotide mutations corresponding to cleavage sites 1 and 2, an AU/GC at position +11/+12 and a UA/CG at +61/+62. M5 harbors a dinucleotide substitution of a GG to AU at position +22/+23. M6 is composed of two dinucleotide substitutions, an AG by UU at +78/+79 in the 3'-end of 79-nt SII and a GC to UU at position +91/+92 just after the 3'-end of 79-nt SII. M6-1 and M6-2 mutants carry one of the two nucleotide substitutions in M6. All mutations are indicated by black arrows. The +1 and +79 positions of the second tandem 79-nt signature sequence of LmjF.36.3810 SIDER2 are indicated by blue arrows. Red arrows indicate cleavage sites (Cl) 1 and 2.

**Fig 3:**
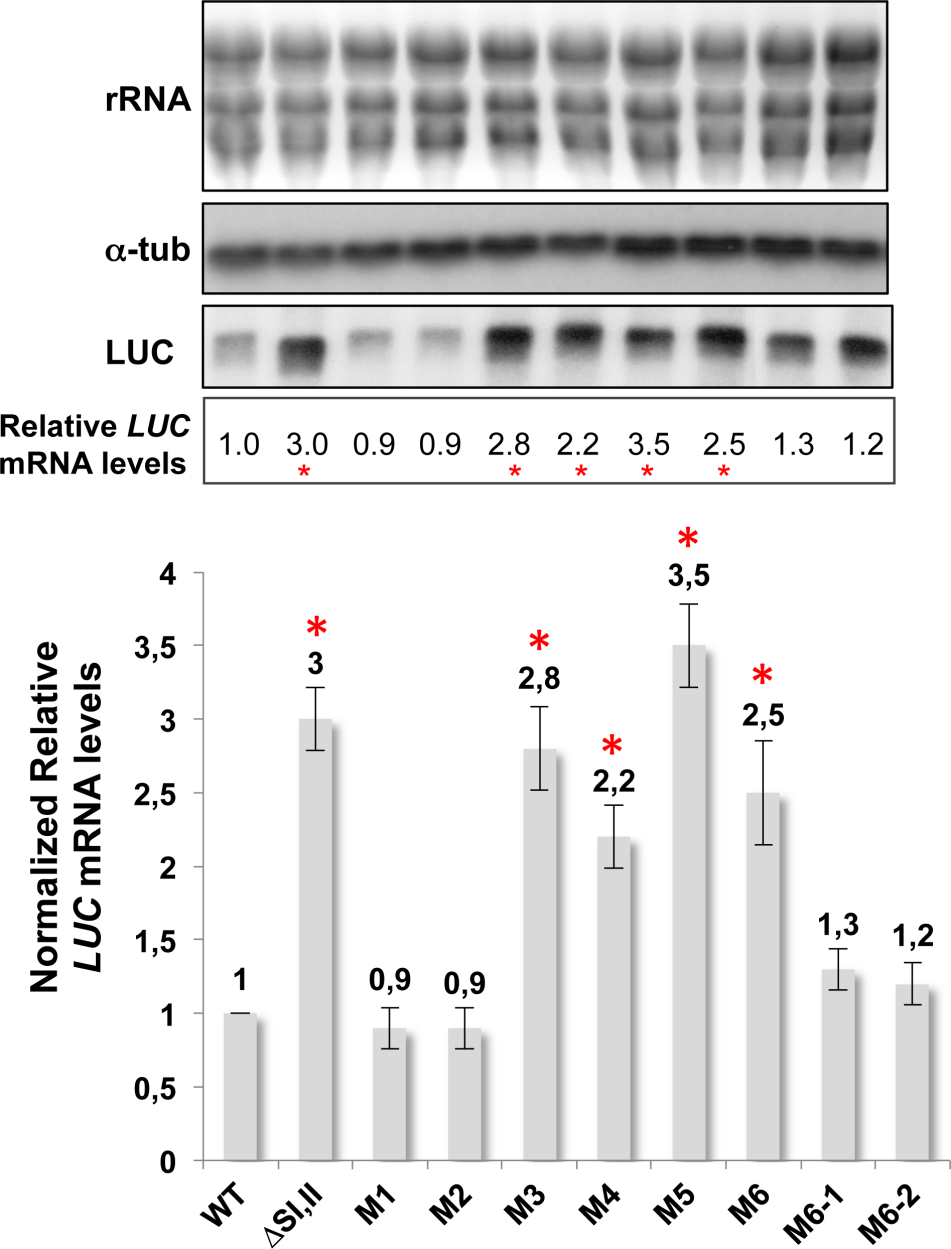
Mutating the cleavage sites and or altering the predicted RNA secondary structure of the second 79-nt SIDER2 signature encompassing the cleavage sites block mRNA decay. Northern blot hybridization of total RNA extracted from recombinant *L*. *major* strains expressing LUC-3810 3'UTR (WT), LUC-3810 3'UTRΔSI+II (ΔSI,II), LUC-3810 3'UTRM1 (M1), LUC-3810 3'UTRM2 (M2), LUC-3810 3'UTRM3 (M3), LUC-3810 3'UTRM4 (M4), LUC-3810 3'UTRM5 (M5), LUC-3810 3'UTRM6 (M6), LUC-3810 3'UTRM6-1 (M6-1) and LUC-3810 3'UTRM6-2 (M6-2). A radiolabeled probe corresponding to the *LUC* gene was used for hybridization to assess changes in *LUC* mRNA expression levels. The alpha-tubulin (α-tub) probe was used as an RNA loading control. An ethidium bromide staining visualizing rRNA is also shown as an additional loading control. The graph displayed in the lower panel represents the normalized fold differences in *LUC* mRNA accumulation in M1 to M6-2 mutants relative to the WT strain. Values were normalized based on RNA loading (*LUC* and alpha-tubulin hybridization signal intensities were quantified using Phosphorimager) and plasmid copy number, varying among the different LUC-transfectants (see also S1 Fig). The mean with standard deviations of three independent experiments is shown here. The asterisk (*) indicates significant changes in *LUC* mRNA accumulation upon normalization.

To better delineate the 79-nt SII primary sequence and secondary structure requirements for efficient mRNA cleavage, we combined RNA structure predictions with extensive mutational analyses (Figs 2 and 3). We used RNAfold [25], one of the core programs of the Vienna RNA package that predicts the minimum free energy (MFE) secondary structure of single-stranded RNA sequences based on the dynamic programming algorithm originally proposed by Zuker and Stiegler [26]. RNA transcripts fold into secondary structures via intricate patterns of base pairing. Equilibrium base-pairing probabilities were calculated via the John McCaskill's partition function (PF) algorithm [27]. The predicted RNA structure of the conserved 79-nt SII within LmjF.36.3810 SIDER2 is organized into 3 hairpin loops. The central hairpin loop is the longest and the most structured harboring both predicted cleavage sites Cl1 and Cl2, which are fold into a single-stranded configuration (Fig 2). We generated eight mutant reporter constructs, of which 7 were within 79-nt SII (M1 to M6-1) and 1 was 12 nucleotides downstream (M6-2) (nucleotides in bold in Fig 1A). According to secondary structure predictions, point mutations M1 (A to C / T to A) and M2 (C to T / A to C) should change the conformation of the upper stem-loop within the central hairpin loop without influencing the structure near Cl1 and Cl2 or elsewhere within 79-nt SII. In M3, one of the two nucleotides in both cleavage sites was substituted (T to C in Cl1 / T to C in Cl2) and based on RNA structure predictions these mutations should severely alter the structure of the central hairpin loop. In M4, both dinucleotides Cl1 and Cl2 were mutated (AT in Cl1 to GC and TA in Cl2 to CG). In M5, two nucleotides were mutated (GG to AT), one of which forms a pair with the first nucleotide (C) of Cl2. This mutation was designed to disrupt the structure of the main stem-loop and the smaller hairpin embedding Cl1 at its end. M6 harbors a combination of AG to TT and GC to TT mutations that were designed to disrupt base-pairing in the predicted hairpin loop structure just after the 3'-end of 79-nt SII and to specifically address the role of this structural part in SIDER2-mediated regulation. Finally, M6-1 and M6-2 were generated from the M6 mutant by introducing dinucleotide substitutions AG to TT or GC to TT individually. These individual mutations should not alter the central hairpin loop structure as well as the 5'-end and 3'-end smaller hairpins surrounding 79-nt SII (Fig 2). All mutant constructs were transfected into *L*. *major* LV39 and total RNA was extracted from stable transfectants and used for Northern blot hybridization to quantify the impact of the mutations and of structural changes on mRNA degradation (Fig 3). Changes in *LUC* mRNA levels in the different 79-nt SII mutants (M1 to M6-2) were calculated relative to wild type SIDER2 sequences and normalized to alpha-tubulin mRNA levels. *LUC* mRNA values were further normalized to the copy number of *LUC*-expressing plasmids due to some variations between the different transfectants (see Materials and Methods and S1 Fig). To quantify relative plasmid copy numbers, DNA from each transfected cell line was analyzed by Southern blot hybridization using a LmjF.36.3810 3'UTR probe recognizing both the *LUC*-expressing plasmids and the genomic copies (S1 Fig).

Our results show that mutations predicted to cause minor conformational changes in the upper stem-loop within the central hairpin loop without affecting Cl1 and Cl2 (M1 and M2), had no effect on *LUC*-3810 decay as mRNA levels were comparable to the wild type 3810 3'UTR RNA (Fig 3). Interestingly, mutations in both cleavage sites (M3 and M4) impeded mRNA decay and resulted in 2.8- and 2.2-fold increase in *LUC* mRNA levels, respectively, which are comparable to those observed upon deletion of the entire 79-nt SII (3-fold) (Fig 3). The effect of M3 was more pronounced, possibly because the predicted central hairpin loop structure was significantly destabilized as inferred from the color code (Figs 2 and 3). Similarly to M3, in M4 where both nucleotides in the dinucleotide cleavage sites were substituted the predicted single-stranded folding within Cl1 and Cl2 was abolished, which may explain blockade in mRNA degradation. The M5 mutant had the highest impact on mRNA stability causing a 3.5-fold increase in *LUC* mRNA abundance (Fig 3). In this mutant, as it was also the case for M3, the predicted central hairpin loop structure was abolished, which may be responsible for the impairment of cleavage and *LUC* mRNA accumulation. Mutations in M6 predicted to destabilize the stem-loop structure formed by the 20-nt following the 3'-end of 79-nt SII caused a 2.5-fold increase in mRNA accumulation, suggesting that this region may also contribute to the folding of 79-nt SII RNA. Finally, individual dinucleotide substitutions in M6-1 and M6-2, which partially disrupt the 3'-end hairpin, had no significant effect on mRNA decay rates (Fig 3).

**Fig 4:**
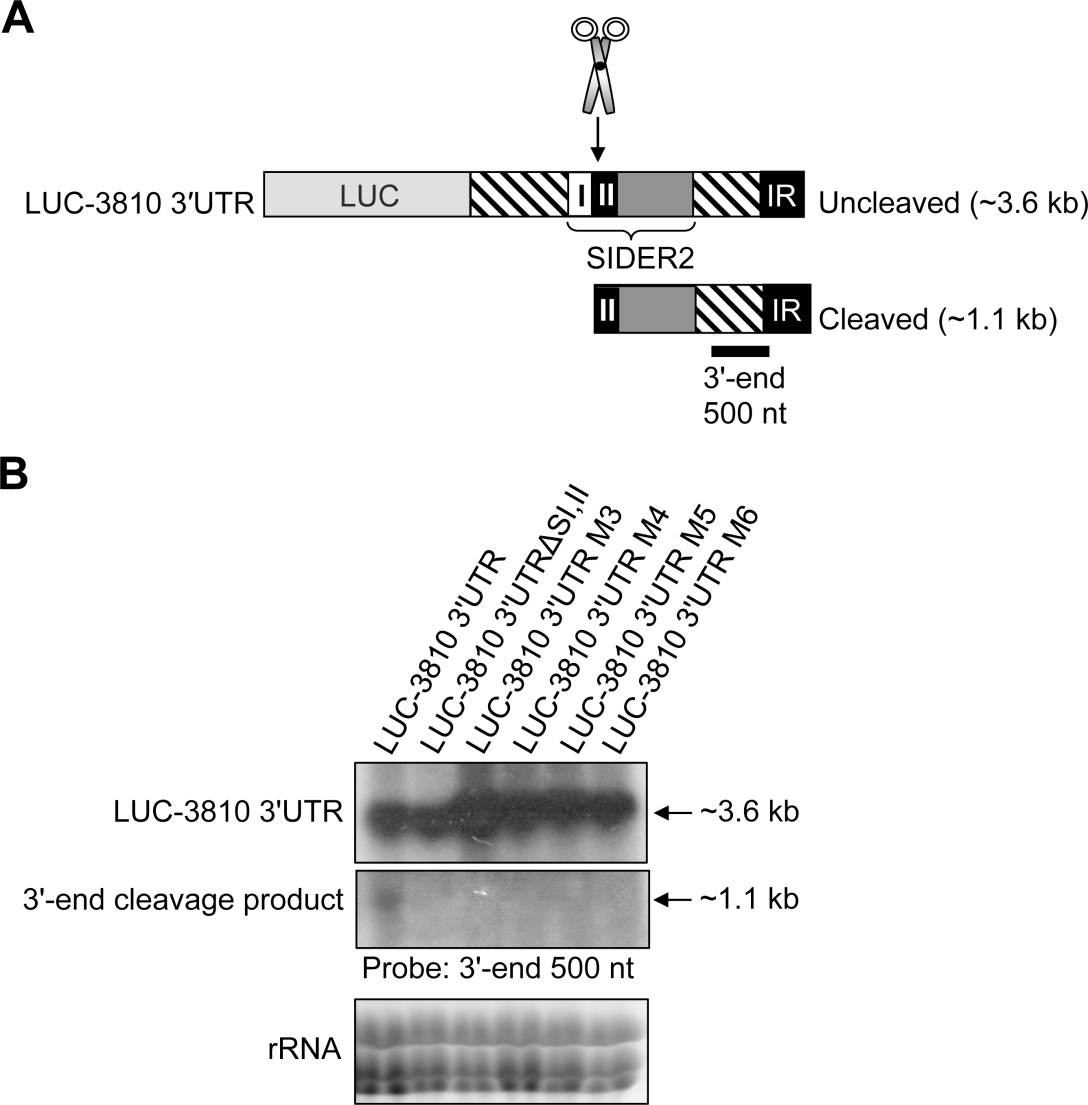
Increased accumulation of mRNAs harboring mutations in the second 79-nt SIDER2 signature is associated with the absence of cleavage products. (A) Schematic representation of the uncleaved and cleaved *L*. *major* LmjF.36.3810 SIDER2-harboring 3'UTR. I and II correspond to the tandem 79-nt signature sequences I and II of 3810 SIDER2, conserved in all SIDER2 retroposons (upper panel). (B) Northern blot hybridization of total RNA isolated from recombinant *L*. *major* LUC-3810 3'UTR, LUC-3810 3'UTRΔSI+II, LUC-3810 3'UTRM3, LUC-3810 3'UTRM4, LUC-3810 3'UTRM5, and LUC-3810 3'UTRM6 strains. The blot was hybridized with a 500 bp radiolabeled probe corresponding to the 3'-end of the LmjF.36.3810 3'UTR, which detects the uncleaved 3.6 kb LUC-3810 3'UTR transcript as well as a cleavage product of ~1.1 kb. An ethidium bromide staining visualizing rRNA is shown here as loading control. Data shown here are representative of two independent experiments yielding comparable results.

Following endonucleolytic cleavage at defined sites within the 79-nt SII *in vivo*, the generated 5'- and 3'-cleavage products can be visualized by different methods [14], including Northern blot hybridization. Although Northern blotting is less sensitive than primer extension and RNase protection assays, it represents the most reliable way to detect and quantify cleavage fragments. A 300 bp DNA probe complementary to the 3'-end of the LmjF.36.3810–3'UTR recognizing both the LUC-3810 3'UTR transcript (~3.6 kb) as well as the 3'-cleavage product of ~1.1 kb was used for hybridization (Fig 4A). Interestingly, the ~1.1 kb 3'-end cleavage product was only detected (after a long exposure of the blot) in LUC-3810 recombinant parasites expressing the wild type 3810–3'UTR, but not in mutants M3, M4, M5 and M6 (Fig 4B) in which mRNA decay was impaired (Fig 3).

Altogether, these results indicate that both predicted cleavage sites are essential for SIDER2-mediated decay, confirming that these dinucleotides are the *bona fide* cleavage sites. They also suggest that the predicted central hairpin loop structure in 79-nt SII encompassing both cleavage sites is key to the regulated SIDER2-mediated mRNA decay process.

### SIDER2 can act autonomously and it is targeted for cleavage outside the 3'UTR context

**Fig 5:**
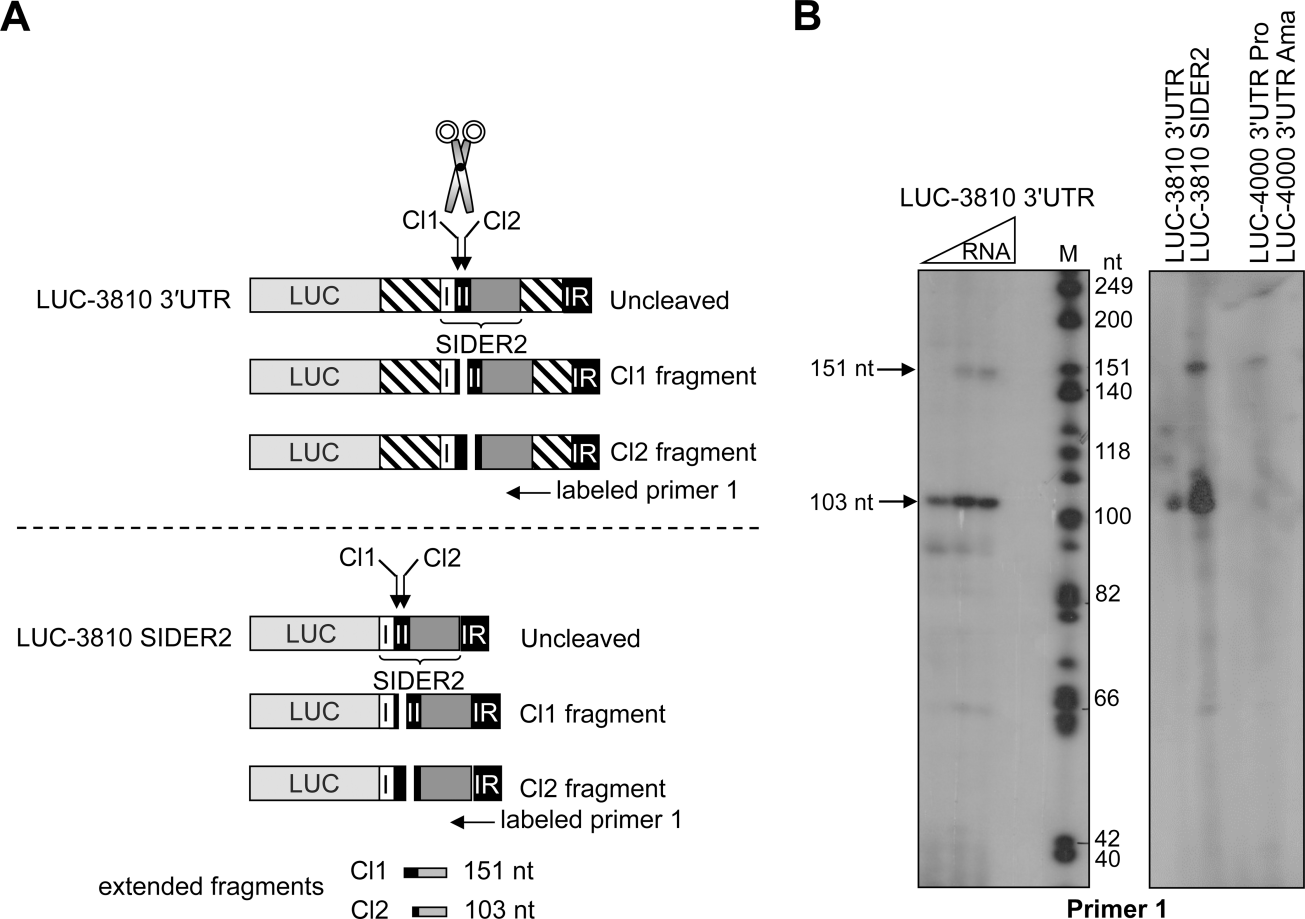
SIDER2 can be targeted for endonucleolytic cleavage when outside its 3'UTR context. (A) Schematic representation of the *in vivo* cleaved *L*. *major* LUC-3810 3′UTR SIDER2-containing mRNA. I and II represent the two conserved 79-nt signature sequences at the 5′end of all SIDER2 retroposons. Depending on the cleavage position within 79-nt SII (2 cleavage sites, Cl1 and Cl2, have been mapped previously in the 3810 SIDER2 element [14]), two fragments of 151-nt (1A) and 103-nt (1B) can be detected by primer extension analysis using a 5′-end labeled primer (P1) complementary to an RNA sequence at ~160-nt downstream. IR, intergenic region downstream of the LmjF.36.3810 3′UTR. (B) Primer extension assay with total RNA extracted from *L*. *major* LUC-3810 3′UTR, LUC-3810 SIDER2 (SIDER2 alone) and from *L*. *infantum* LUC-4000 3′UTR SIDER2-containing mRNAs using primer 1. Two specific extension fragments of 151-nt and 103-nt were resolved on 8% denaturating acrylamide gel corresponding to both cleavage sites 1 and 2, respectively. Their sizes were estimated with a radiolabeled DNA marker (M) are in agreement with previous results [14]. For the *L*. *infantum* LUC-4000 3′UTR SIDER2-containing mRNA, promastigotes (Pro) and axenic amastigotes (Ama) were used for primer extension to determine whether preferential decay of the *L*. *infantum* LinJ.36.4000 mRNA in promastigotes was correlated to stage-regulated cleavage. The primer extension data shown here are representative of at least 3 independent experiments yielding comparable results.

We have shown previously that a SIDER2 retroposon (~550 bp) when placed downstream of a *LUC* reporter gene was sufficient to confer rapid mRNA turnover both in *L*. *major* and *L*. *infantum* [13]. Here, we investigated whether SIDER2 sequences outside their 3'UTR context were also targeted for endonucleolytic cleavage. For this, we carried out primer extension (PE) analysis to visualize cleavage fragments, as successfully done previously [14]. We used primer 1 that proved to be specific and sensitive in detecting cleavage fragments within the 79-nt SII of the LmjF.36.3810 3'UTR [14] (Fig 5A). Interestingly, PE analysis of total RNA from parasites expressing the LUC-3810 SIDER2 construct where only SIDER2 sequences were fused to *LUC* yielded two cleavage products of 103-nt and 151-nt (Fig 5B, right panel), similarly to the full length LUC-3810 3'UTR (Fig 5B, left panel) and to previous results [14]. The higher signal intensity of LUC-3810 SIDER2 RNA is due to the higher plasmid copy number in this recombinant parasite (data not shown). This result confirms that destabilization of LUC-3810 SIDER2 mRNA [13] is genuine and results from endonucleolytic cleavage within 79-nt SII. Moreover, it suggests that SIDER2 can function autonomously in promoting rapid mRNA decay and does not require flanking 3'UTR sequences. One reason for this autonomy might be the fact that the 79-nt SII regulatory region folds into a distinct structure (S2 Fig) as suggested by secondary structure RNA predictions and our mutational analyses.

### Stage-specific SIDER2 inactivation correlates with the absence of endonucleolytic cleavage

**Fig 6:**
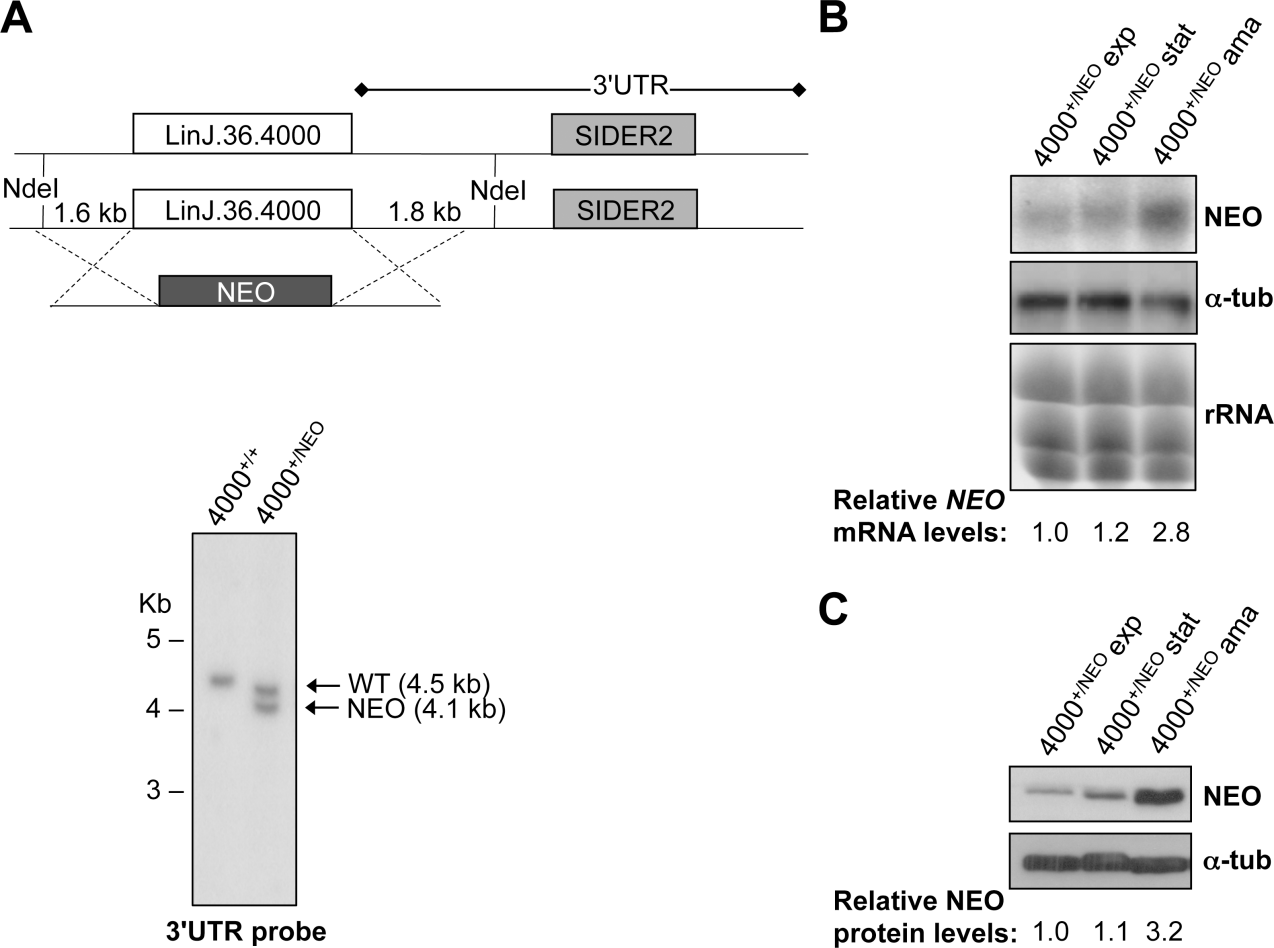
Stage-specific regulation of SIDER2-containing mRNAs does not depend on the genomic context and is mediated solely by SIDER2 sequences within 3'UTR. (A) Genomic integration of the neomycin phosphotransferase gene (*NEO*) into the *L*. *infantum* LinJ.36.4000 locus by homologous recombination through 500 bp homologous sequences flanking the 5'- and 3'-ends of LinJ.36.4000 gene (upper panel). Replacement of one LinJ.36.4000 genomic copy by the *NEO* gene was confirmed by Southern blot hybridization of genomic DNA digested with NdeI using a radiolabeled DNA probe recognizing the first 1 kb of the LinJ.36.4000 3'UTR (lower panel). The slight shift of the wild type (WT) 4.5 kb band in 4000^+/NEO^ is due to differences in migration. (B) Northern blot of total RNA extracted from *L*. *infantum* exponential (exp), stationary (stat) and axenic amastigote (ama) cultures of LinJ.36.4000^+/NEO^ parasites hybridized with a radiolabeled *NEO*-specific probe. Hybridization signals were quantified by the ImageQuant 5.2 software and values were normalized to the ethidium bromide stained rRNA and calculated relatively to the exponentially grown LinJ.36.4000^+/NEO^ promastigotes. (C) Western blot analysis of total protein lysates from LinJ.36.4000^+/NEO^ exponentially grown and stationary promastigotes and also axenic amastigotes. The blot was incubated with an anti-NEO antibody and subsequently with an anti-α-tubulin antibody for normalization purposes. The resulting signals were quantified using the ImageQuant 5.2 software and normalized values were expressed relatively to the exponentially grown LinJ.36.4000^+/NEO^ promastigotes. Data shown here are representative of two independent experiments with similar results.

SIDER2-mediated mRNA decay can be regulated in a stage-specific manner [13]. For example, the SIDER2 element within the 3'UTR of LinJ.36.4000 mRNA (4000), the *L*. *infantum* ortholog of LmjF.36.3810 (3810), causes a rapid turnover of the *4000* mRNA in promastigotes but in amastigotes the same transcript accumulates, suggesting that SIDER2 is inactive in this life stage [13]. To address the question of how SIDER2 is inactivated in amastigotes, we first investigated the regulation of LinJ.36.4000 decay within its genomic context. For this, we replaced one copy of the LinJ.36.4000 coding region with the neomycin phosphotransferase (*NEO*) gene (Fig 6A, upper panel). Integration of the *NEO* coding region was confirmed by Southern blotting (Fig 6A, lower panel) and also by PCR analysis (not shown). Expression levels of the *NEO* mRNA harboring the LinJ.36.4000 3’UTR were subsequently quantified by northern blot hybridization in *L*. *infantum* exponential and stationary promastigotes as well as in fully differentiated axenic amastigotes. Our results show a 2.8-fold increase in the *NEO* mRNA abundance in amastigotes compared with exponential and stationary-phase promastigotes (Fig 6B). *NEO* mRNA levels correlate with protein expression levels (Fig 6C). These results are in agreement with our previous experiments using the episomal LUC reporter system and endogenous SIDER2-containing transcripts [13, 19]. Altogether, these results suggest that increased accumulation of LinJ.36.4000 mRNA in amastigotes does not depend on its genomic context (e.g. effect of the open reading frame on mRNA stability) or on alternative splicing (e.g. 5'UTR sequence effect) but solely on the inactivation of SIDER2 present within its 3'UTR.

Next, we investigated whether inhibition of 4000 SIDER2-mediated decay in amastigotes is due to impaired endonucleolytic cleavage, the initial step in SIDER2-mediated decay [14]. To detect cleavage fragments, we performed PE assays using primer 1 and total RNA both from *L*. *infantum* promastigotes and amastigotes expressing LUC-4000 3'UTR. This analysis revealed two cleavage fragments of 103-nt and 151-nt in promastigotes similarly to the orthologous LUC-3810 SIDER2 in *L*. *major* (Fig 5B) and in agreement with the rapid degradation of *LUC*-4000 transcript in this life stage [13]. In contrast, no cleavage products were detected using similar amounts of RNA isolated from *L*. *infantum* LUC-4000 3'UTR amastigotes (Fig 5B). This suggests that SIDER2 is not longer functional, cleavage is hence impaired, and transcripts can accumulate in this life stage.

## Discussion

We have reported previously a novel mechanism of regulated mRNA decay in *Leishmania* involving **S**hort **I**nterspersed **D**egenerate **R**etroposons of the SIDER2 subfamily, located largely within 3'UTRs of *Leishmania* transcripts. We have shown that the second tandem 79-nt hallmark signature sequence (79-nt SII), conserved among all SIDER2 retroposons, is essential for regulation and that it mediates rapid mRNA turnover through endonucleolytic cleavage [13, 14]. In this study, we provide new insights into the sequence and structural requirements for efficient mRNA cleavage and degradation using mutational and *in silico* RNA structural studies to further delineate the *cis*-acting region within 79-nt SII.

We show that single or double nucleotide substitutions within both predicted cleavage sites (Cl1 and Cl2) in the LmjF.36.3810 SIDER2 element [14], N-pyrimidine (AU) or pyrimidine-pyrimidine (CU), similarly blocked mRNA degradation, indicating that both cleavage sites are essential for SIDER2-mediated mRNA decay and hence confirming that these dinucleotides are the *bona fide* cleavage sites. Although the predicted changes in the LmjF.36.3810 SIDER2 79-nt SII RNA secondary structure when mutating one or both nucleotides in each dinucleotide cleavage site were not of the same magnitude, Cl1 and Cl2 folded into a double-stranded structure and were no longer single-stranded as in the wild type. These structural changes may have also contributed to the pronounced effect in blocking mRNA degradation. In fact, several classes of RNA-binding proteins, including proteins with an RNA-recognition motif (RRM), double-stranded RNA-binding motif (ds-RBD) like the Staufen 1 (STAU1) proteins mediating mRNA decay [28], as well as zinc finger motifs have been shown to recognize not only the sequence but also the structure of the mRNA [29–31]. Although *in silico* predicted RNA structures suggest that both cleavage sites are in a single-stranded conformation, it would be important to determine the structure of the 79-nt SII RNA within SIDER2 elements using nuclear magnetic resonance spectroscopy (NMR) or related technologies. Mutations predicted to disrupt the central hairpin loop structure embedding Cl2 and Cl1 sites had the most dramatic impact on mRNA decay, resulting in the highest increase in mRNA accumulation. In addition, mutations designed to specifically disrupt base-pairing in the predicted hairpin structure at the 3'-end of 79-nt SII also affected the decay process. Altogether, these data suggest that RNA secondary structure of the conserved 79-nt SII within SIDER2, especially in the vicinity of the cleavage sites, may be crucial for an efficient mRNA decay. In *Trypanosoma* spp., it was recently shown that the first signature sequence of the potentially functional L1*Tc*/*ingi* clade retrotransposons, the ancestors of SIDERs, forms a Hepatitis Delta Virus (HDV)-like ribozyme [32]. Indeed, the first signature of SIDER2 elements seems to fit to the HDV-like ribozyme folding of L1TcRz, however, the 79-nt SII does clearly not (Jean-Pierre Perreault, University of Sherbrooke, personal communication). Moreover, *in vitro* ribozyme function was not detectable in the 3′UTR region of *Leishmania* SIDER2-harboring mRNAs undergoing degradation [14, 33] and recent work suggests a co-translational mechanism for SIDER2-mediated mRNA decay, supporting the involvement of protein factors in this process [34]. In line with this work, using in vivo tethering assays, we identified RNA-binding proteins that once tethered to a SIDER2-harboring 3′UTR enhance its degradation (Hiva Azizi *et al*., manuscript in preparation).

We have reported previously that a SIDER2 element outside its 3'UTR context was able to promote rapid mRNA degradation to levels comparable with a 3'UTR bearing a SIDER2 element [13]. Here, we provide further evidence, using primer extension assays, that degradation of a reporter transcript fused to a SIDER2 element only (e.g. LmjF.36.3810 SIDER2) is also initiated by endonucleolytic cleavage and that cleavage occurs exactly at the same nucleotide positions within 79-nt SII as previously described for a reporter transcript with the full-length SIDER2-harboring 3'UTR [14]. This confirms that SIDER2 alone is sufficient for initiating mRNA cleavage and subsequent degradation without the contribution of neighboring 3'UTR sequences [13]. This might be possible because the 79-nt SII folds into a distinct stable structure, as suggested by RNA secondary structure predictions using RNAfold. This structure could be recognized by an endoribonuclease and associated decay factors.

Our previous studies have demonstrated that SIDER2-mediated mRNA decay is regulated in a stage-specific manner. For the *L*. *infantum* LinJ.36.4000 gene, the ortholog of LmjF.36.3810, encoding an aminomethyltransferase of the glycine cleavage complex [19], we showed that SIDER2 was able to promote rapid turnover of the mRNA in promastigotes but not in amastigotes [13]. Increasing evidence in the literature supports that during the differentiation process, *Leishmania* switches from glycolysis to amino acid catabolism and that proteins involved in protein degradation and amino acid import are significantly upregulated in amastigotes [18, 35, 36]. These observations are in line with the increased expression of aminomethyltransferase in *L*. *infantum* amastigotes. Here, we show that stage-specific accumulation of LinJ.36.4000 mRNA in amastigotes correlates with the absence of endonucleolytic cleavage, which suggests that SIDER2 is inactive. Additional experiments using an integrated reporter system where the LinJ.36.4000 genomic copy was replaced by the neomycin phosphotransferase (*NEO*) gene, revealed a selective *NEO* mRNA degradation in promastigotes but increased accumulation in amastigotes as a result of SIDER2 inactivation. A SIDER2 element may loose its ability to promote mRNA decay because of changes in the RNA structure probably caused by an increase in temperature during promastigote to amastigote differentiation. These structural changes could alter the binding of specific trans-acting regulators to SIDER2 RNA or alternatively allow stage-regulated factors to interact with the RNA and block access of the decay complex.

In summary, our results demonstrate that both predicted dinucleotide cleavage sites within the conserved 79-nt SII of SIDER2 elements are essential for SIDER2-mediated mRNA decay and further support that SIDER2-harboring transcripts are targeted for degradation by endonucleolytic cleavage. Furthermore, our in silico analysis underscores the role of RNA structure, especially in the vicinity of the two cleavage sites, in the decay process. Although the studies here focused on two selected SIDER2 elements in *L*. *major* as well as in *L*. *infantum*, our findings could possibly apply to a larger number of SIDER2 elements given the high conservation of the 79-nt SII. Overall, these studies provide additional insights into the complex mechanism of SIDER2-mediated mRNA decay in *Leishmania*.

## Supporting information

S1 Table Primers used in this study. (DOCX)

S1 Fig Plasmid copy number estimates in *L*. *major* recombinant strains stably expressing LUC-3810 3'UTR or LUC-3810 3'UTRΔSI+II and or a variety of LUC-3810 3'UTR mutants within the second 79-nt hallmark sequence (signature II) of SIDER2 retroposons. Total DNA was extracted from *L*. *major* LUC-3810 3'UTR (WT), LUC-3810 3'UTRΔSI+II ΔSI,II), LUC-3810 3'UTRM1 (M1), LUC-3810 3'UTRM2 (M2), LUC-3810 3'UTRM3 (M3), LUC-3810 3'UTRM4 (M4), LUC-3810 3'UTRM5 (M5), LUC-3810 3'UTRM6 (M6), LUC-3810 3'UTRM6-1 (M6-1) and LUC-3810 3'UTRM6-2 (M6-2), digested with NdeI and analyzed by Southern blot hybridization using a radiolabeled DNA probe complementary to first 1kb region of the 3810–3'UTR. This probe recognizes both the LmjF.36.1830 genomic locus (2 copies as this *L*. *major* strain is diploid for chr 36; a ~4 kb band) and the linearized episomal vector (~8 kb band). Quantification of the hybridization intensity signals was carried out using PhosphorImager. The ratio of the plasmid signal vs. the genomic signal provides an estimate of the copy number of the episomal *LUC*-expressing vector in each transfectant. These values are shown in the upper graph.
(TIF)

S2 Fig Predicted secondary structure of the LmJF.36.3810 SIDER2 RNA. RNA predictions were carried using the RNAfold web server according to the Minimum Free Energy (MFE). The 79-nt signature II (SII) sequence of LmJF.36.3810 SIDER2 is surrounded by a red box. Colors in the structures indicate base-pairing probabilities according to a scale of 0 to 1 (0: magenda; 1: red).
(TIF)
