# Supplementary material for: RNA secondary structure and nucleotide composition of the conserved hallmark sequence of Leishmania SIDER2 retroposons are essential for endonucleolytic cleavage and mRNA degradation
Source: PLoS One. 2017 Jul 13;12(7):e0180678. doi: 10.1371/journal.pone.0180678 (PMC5509151; doi:10.1371/journal.pone.0180678)
Supplement: S1 Table — (DOCX) [file pone.0180678.s001.docx]

**S1 Table. Primers used in this study.**

| **Mutations introduced in the second 79-nt signature II sequence (79-nt SII) of LmjF.36.3810 SIDER2** | | | | |
| --- | --- | --- | --- | --- |
|  | **Upstream Fragment** | | **Downstream Fragment** | |
| 3'UTR-3810ΔCl1 | 3ʹUTR-3810 F BamHI | 5'cgc**GGATCC**AACGGCCGTGCTCCGACGAACACAGGC3' | SIDER2-3810Cl1 F | 5'TGCGAGGTGCCGAGCCACTTCTCGTGGTGAAAGGG3' |
|  | SIDER2-3810ΔCl1 R | 5'AGTGGCTCGGCACCTCGCAAAAGGGGAGGAGGCCGC3' | 3ʹUTR-3810 R HindIII | 5'ccc**AAGCTT**CGTGGGAAGAGGGCGAGCAGAGAGAGC3' |
| 3'UTR-3810ΔCl2 | 3ʹUTR-3810 F BamHI | 5'cgc**GGATCC**AACGGCCGTGCTCCGACGAACACAGGC3' | SIDER3810ΔCl2 F | 5'TGCGAGGTGCCGAGCCACTTCTCGTGGTGAAAGGG3' |
|  | SIDER3810ΔCl2 R | 5'AGTGGCTCGGCACCTCGCAAAAGGGGAGGAGGCCGC3' | 3ʹUTR-3810 R HindIII | 5'ccc**AAGCTT**CGTGGGAAGAGGGCGAGCAGAGAGAGC3' |
| 3'UTR-3810 M1 | 3ʹUTR-3810 F BamHI | 5'cgc**GGATCC**AACGGCCGTGCTCCGACGAACACAGGC3' | SIDER2-3810 M1 F | 5'GGGCCAAGCACCTACGGCGAGG3' |
|  | SIDER2-3810M1 R | 5'CGCCGTAGGTGCTTGGCCCTTTCACCtCGAGAAGgGGCTCGGCACCTCGCAGGGG3' | 3ʹUTR-3810 R HindIII | 5'ccc**AAGCTT**CGTGGGAAGAGGGCGAGCAGAGAGAGC3' |
| 3'UTR-3810 M2 | 3ʹUTR-3810 F BamHI | 5'cgc**GGATCC**AACGGCCGTGCTCCGACGAACACAGGC3' | SIDER2-3810 M2 F | 5'GGGCCAAGCACCTACGGCGAGG3' |
|  | SIDER2-3810M2 R | 5'CGCCGTAGGTGCTTGGCCCTgTCACCACGAaAAGTGGCTCGGCACCTCGCAGGGG3' | 3ʹUTR-3810 R HindIII | 5'ccc**AAGCTT**CGTGGGAAGAGGGCGAGCAGAGAGAGC3' |
| 3'UTR-3810 M3 | 3ʹUTR-3810 F BamHI | 5'cgc**GGATCC**AACGGCCGTGCTCCGACGAACACAGGC3' | SIDER2-3810 M3 F | 5'CGGCGAGGGGAAGTCAGTGCGATTCATCGC3' |
|  | SIDER2-3810 M3 R | 5'GACTTCCCCTCGCCGTgGGTGCTTGGCCCTTTCACCACGAGAAGTGGCTCGGCACCTCGCAGGGGgTCGAAAGGGGAGGAGG3' | 3ʹUTR-3810 R HindIII | 5'ccc**AAGCTT**CGTGGGAAGAGGGCGAGCAGAGAGAGC3' |
| 3'UTR-3810 M4 | 3ʹUTR-3810 F BamHI | 5'cgc**GGATCC**AACGGCCGTGCTCCGACGAACACAGGC3' | SIDER2-3810 M4 F | 5'CGGCGAGGGGAAGTCAGTGCGATTCATCGC3' |
|  | SIDER2-3810 M4 R | 5'CTGACTTCCCCTCGCCGcgGGTGCTTGGCCCTTTCACCACGAGAAGTGGCTCGGCACCTCGCAGGGGgcCGAAAGGGGAGGAGGCC3' | 3ʹUTR-3810 R HindIII | 5'ccc**AAGCTT**CGTGGGAAGAGGGCGAGCAGAGAGAGC3' |
| 3'UTR-3810 M5 | 3ʹUTR-3810 F BamHI | 5'cgc**GGATCC**AACGGCCGTGCTCCGACGAACACAGGC3' | SIDER2-3810 M5 F | 5'GCCGAGCCACTTCTCGTGGTGAAAGGGC3' |
|  | SIDER2-3810 M5 R | 5'CACCACGAGAAGTGGCTCGGCAatTCGCAGGGGATCGAAAGGGGAG3' | 3ʹUTR-3810 R HindIII | 5'ccc**AAGCTT**CGTGGGAAGAGGGCGAGCAGAGAGAGC3' |
| 3'UTR-3810 M6 | 3ʹUTR-3810 F BamHI | 5'cgc**GGATCC**AACGGCCGTGCTCCGACGAACACAGGC3' | SIDER2-3810 M6 F | 5'CACTGACACCGGCAGTCAGGTCCTGG3' |
|  | SIDER2-3810 M6 R | 5'GACTGCCGGTGTCAGTGaaGATGAATCGCAaaGACTTCCCCTCGCCGTAG3' | 3ʹUTR-3810R HindIII | 5'ccc**AAGCTT**CGTGGGAAGAGGGCGAGCAGAGAGAGC3' |
| 3'UTR-3810M6-1 | 3ʹUTR-3810 F BamHI | 5'cgc**GGATCC**AACGGCCGTGCTCCGACGAACACAGGC3' | SIDER2-3810 M6-1 F | 5'TGCGATTCATCGCCACTGACA3' |
|  | SIDER2-3810 M6-1 R | 5'TGTCAGTGGCGATGAATCGCAaaGACTTCCCCTCGCCGT3' | 3ʹUTR-3810 R HindIII | 5'ccc**AAGCTT**CGTGGGAAGAGGGCGAGCAGAGAGAGC3' |
| 3'UTR-3810M6-2 | 3ʹUTR-3810 F BamHI | 5'cgc**GGATCC**AACGGCCGTGCTCCGACGAACACAGGC3' | 3810-M6-2 F1 | 5'CATCTTCACTGACACCGGCAG3' |
|  | SIDER2-3810 M6-2 R | 5'CTGCCGGTGTCAGTGaaGATGAATCGCACTGACTTC3' | 3ʹUTR-3810 R HindIII | 5'ccc**AAGCTT**CGTGGGAAGAGGGCGAGCAGAGAGAGC3' |
| **Primers used to integrate the *NEO* gene into the LinJ.36.4000 genomic locus** | | | | |
| PCR Fragment | Primer Name | Primer sequences | | |
| 5'UTR of LinJ.36.4000 | 5'UTR4000 F | 5’GGTGACCGGCGCAGGTGTGCA3’ | | |
|  | 5'UTR4000 R | 5’CAATCCATCTTGTTCAATCATGGCTGCCGCGGGAAAGGAGT3’ | | |
| NEO ORF | NEO F | 5’ATGATTGAACAAGATGGATTG3’ | | |
|  | NEO R | 5’TCAGAAGAACTCGTCAAGAAG3’ | | |
| 3'UTR of LinJ.36.4000 | 3'UTR4000 F | 5’CTTCTTGACGAGTTCTTCTGAAACGGCCGTGCTCCGACA3’ | | |
|  | 3'UTR4000 R | 5’GTCTGTACGAGTGTGCGCTTGT3’ | | |
| **Primers used for primer extension assays** | | | | |
| 3810-P1  4000-P1 | | 5’ -GCACAGGCCTGCTCACTGTC-3’  5’-AATGGCCCGAGCATATTGG -3’ | | |
