# Supplementary figures and images for: RNA secondary structure and nucleotide composition of the conserved hallmark sequence of Leishmania SIDER2 retroposons are essential for endonucleolytic cleavage and mRNA degradation
Source: PLoS One. 2017 Jul 13;12(7):e0180678. doi: 10.1371/journal.pone.0180678 (PMC5509151; doi:10.1371/journal.pone.0180678)

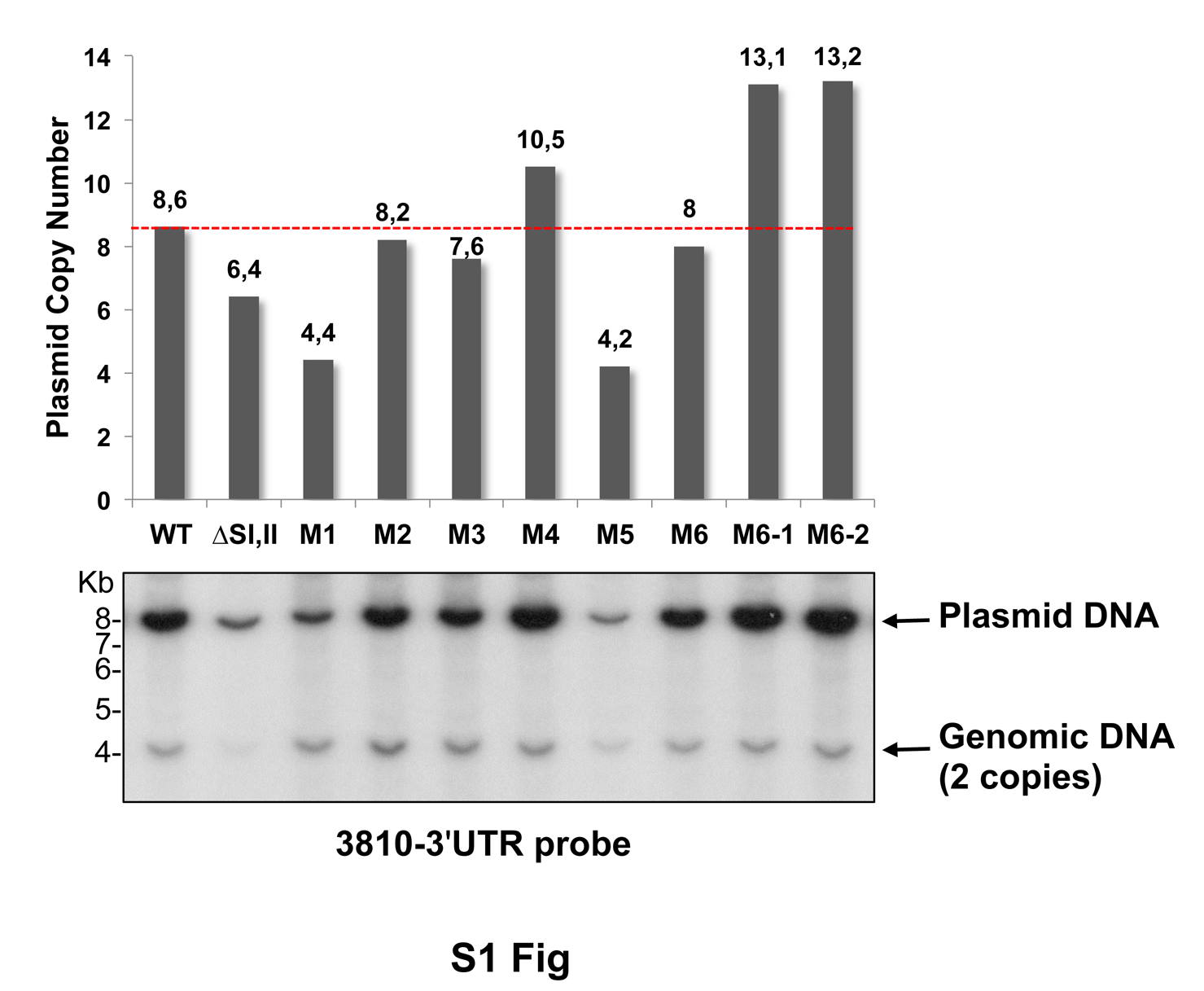

Supplement: S1 Fig — Total DNA was extracted from L. major LUC-3810 3'UTR (WT), LUC-3810 3'UTRΔSI+II ΔSI,II), LUC-3810 3'UTRM1 (M1), LUC-3810 3'UTRM2 (M2), LUC-3810 3'UTRM3 (M3), LUC-3810 3'UTRM4 (M4), LUC-3810 3'UTRM5 (M5), LUC-3810 3'UTRM6 (M6), LUC-3810 3'UTRM6-1 (M6-1) and LUC-3810 3'UTRM6-2 (M6-2), digested with NdeI and analyzed by Southern blot hybridization using a radiolabeled DNA probe complementary to first 1kb region of the 3810–3'UTR. This probe recognizes both the LmjF.36.1830 genomic locus (2 copies as this L. major strain is diploid for chr 36; a ~4 kb band) and the linearized episomal vector (~8 kb band). Quantification of the hybridization intensity signals was carried out using PhosphorImager. The ratio of the plasmid signal vs. the genomic signal provides an estimate of the copy number of the episomal LUC-expressing vector in each transfectant. These values are shown in the upper graph. (TIF) [file pone.0180678.s002.tif]

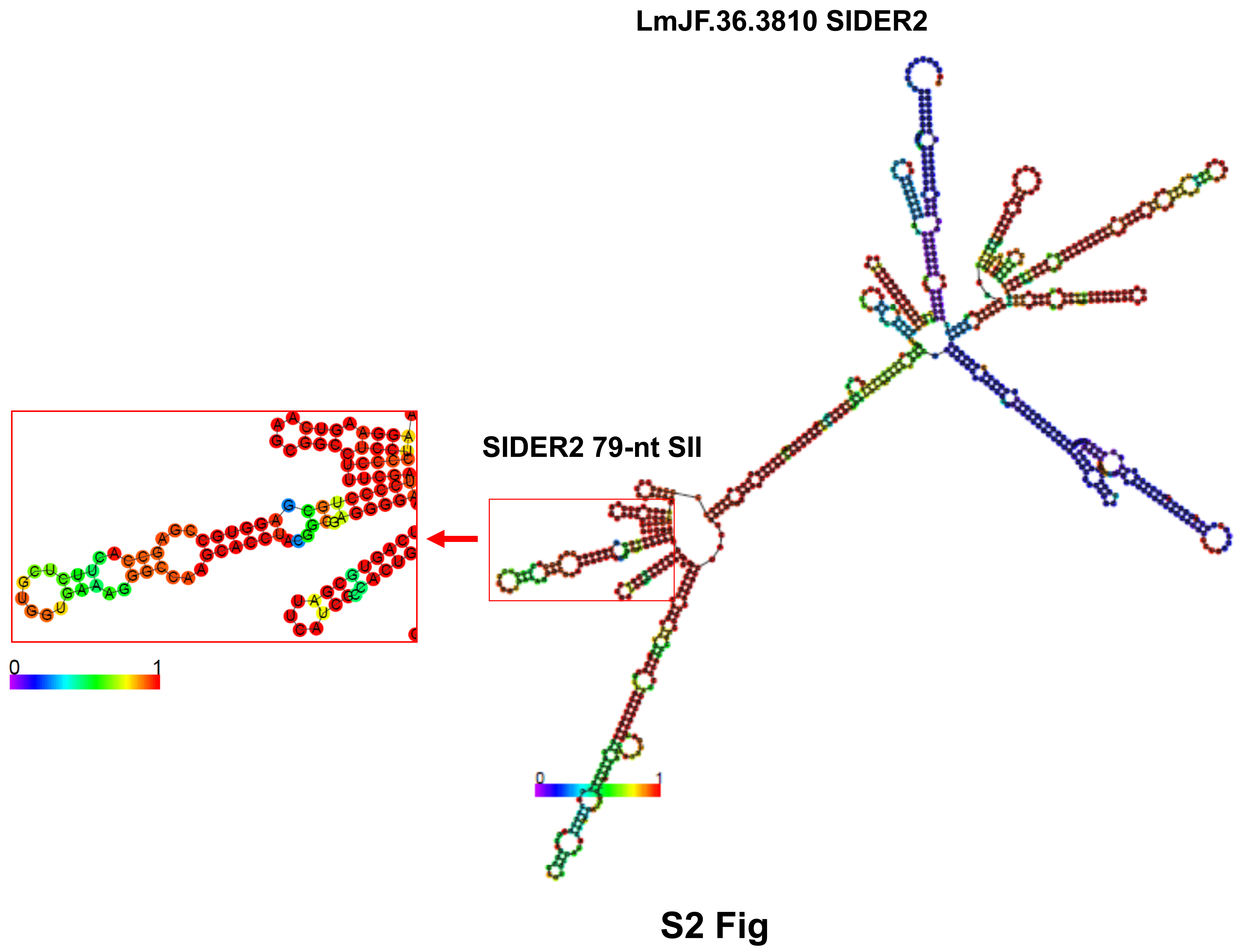

Supplement: S2 Fig — RNA predictions were carried using the RNAfold web server according to the Minimum Free Energy (MFE). The 79-nt signature II (SII) sequence of LmJF.36.3810 SIDER2 is surrounded by a red box. Colors in the structures indicate base-pairing probabilities according to a scale of 0 to 1 (0: magenda; 1: red). (TIF) [file pone.0180678.s003.tif]
